# Supplementary material for: Nutritional Composition, In Vitro Starch Digestibility and Antioxidant Activities of Composite Flour Made from Wheat and Mature, Unripe Pawpaw (Carica papaya) Fruit Flour
Source: Nutrients. 2022 Nov 14;14(22):4821. doi: 10.3390/nu14224821 (PMC9697140; doi:10.3390/nu14224821)
Supplement: Supplementary file 1 [file nutrients-14-04821-s001.zip › nutrients-2001667-supplementary.pdf]

Table S1. In-vitro starch digestibility of composite flour made from wheat and mature unripe pawpaw fruit flour.

Samples	RSD	SDS	RS
100% Wheat flour	96.33±1.21	2.44±0.10	1.23±0.10
90 WF + 10 MUPFF	79.91±0.92	11.56±0.32	8.53±0.10
80 WF + 20 MUPFF	66.73±0.56	21.34±0.33	11.93±0.25
70 WF + 30 MUPFF	52.87±0.62	25.22±0.41	21.91±0.23
60 WF + 40 MUPFF	40.53±0.50	31.96±0.45	27.51±0.21
50 WF + 50 MUPFF	29.22±0.32	34.67±0.34	36.11±0.24

Values are means ± standard deviations of replicate determinations (n=3). Mean values with the same letter in the same column are not significantly ($p>0.05$) different. WF is wheat flour; MUPFF is Mature unripe pawpaw fruit flour.

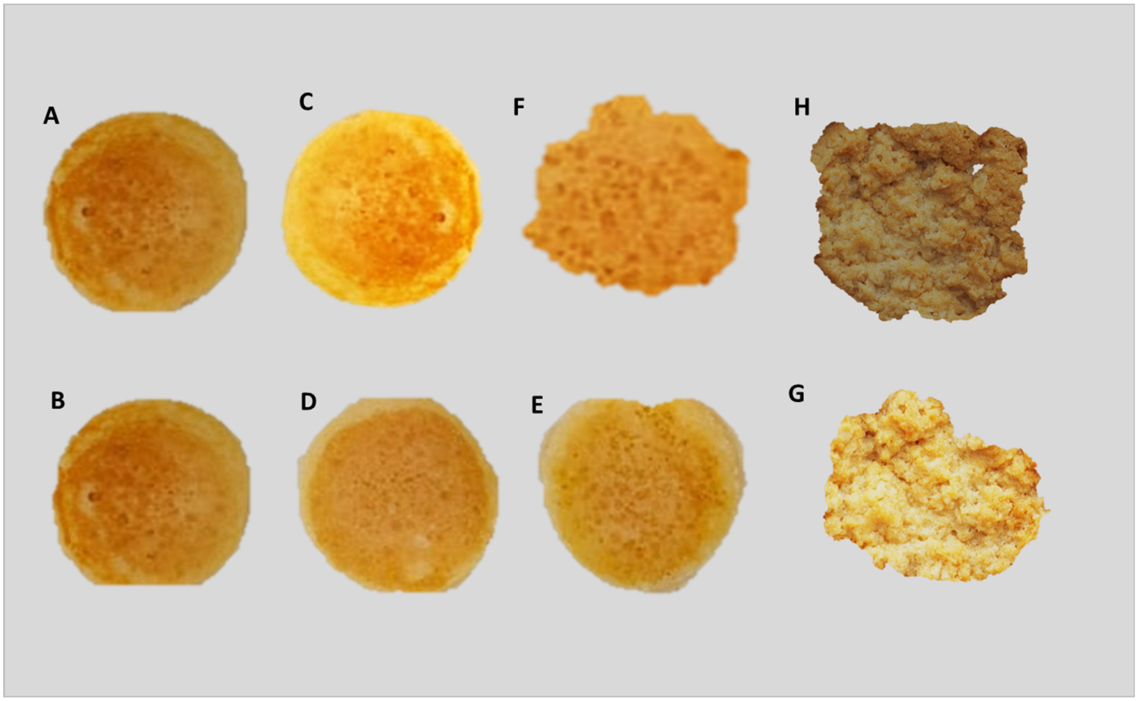

Figure S1. Pictures of cookies baked from composite flour from wheat and mature unripe pawpaw fruit flour (MUPF).
